# Efficacy and safety of paricalcitol in children with stages 3 to 5 chronic kidney disease

**DOI:** 10.1007/s00467-017-3579-6

**Published:** 2017-03-22

**Authors:** Nicholas J. A. Webb, Gary Lerner, Bradley A. Warady, Katherine M. Dell, Larry A. Greenbaum, Gema Ariceta, Bernd Hoppe, Peter Linde, Ho-Jin Lee, Ann Eldred, Matthew B. Dufek

**Affiliations:** 1 Department of Paediatric Nephrology and NIHR/Wellcome Trust Clinical Research Facility, Manchester Academic Health Science Centre, Royal Manchester Children’s Hospital, University of Manchester, Oxford Road, Manchester, M13 9WL UK; 2 Pediatric Nephrology, Keck School of Medicine–Children’s Hospital Los Angeles, Los Angeles, CA USA; 3 Division of Pediatric Nephrology, Children’s Mercy Hospital, Kansas City, MO USA; 4 Center for Pediatric Nephrology, Cleveland Clinic Children’s, Case Western Reserve University, Cleveland, OH USA; 5 Emory School of Medicine and Children’s Healthcare of Atlanta, Atlanta, GA USA; 6 Pediatric Nephrology, University Hospital Vall d’Hebron, Universitat Autonoma de Barcelona, Barcelona, Spain; 7 University Hospital Bonn, Bonn, Germany; 8 AbbVie Inc., North Chicago, IL USA

**Keywords:** Secondary hyperparathyroidism, Paricalcitol, Chronic kidney disease–mineral and bone disorder, Pediatric, Hypercalcemia

## Abstract

**Background:**

Elevated intact parathyroid hormone (iPTH) levels can contribute to morbidity and mortality in children with chronic kidney disease (CKD). We evaluated the pharmacokinetics, efficacy, and safety of oral paricalcitol in reducing iPTH levels in children with stages 3–5 CKD.

**Methods:**

Children aged 10–16 years with stages 3–5 CKD were enrolled in two phase 3 studies. The stage 3/4 CKD study characterized paricalcitol pharmacokinetics and compared the efficacy and safety of paricalcitol with placebo followed by an open-label period. The stage 5 CKD study evaluated the efficacy and safety of paricalcitol (no comparator) in children with stage 5 CKD undergoing dialysis.

**Results:**

In the stage 3/4 CKD study, mean peak plasma concentration and area under the time curve from zero to infinity were 0.13 ng/mL and 2.87 ng•h/((or ng×h/))mL, respectively, for 12 children who received 3 μg paricalcitol. Thirty-six children were randomized to paricalcitol or placebo; 27.8% of the paricalcitol group achieved two consecutive iPTH reductions of ≥30% from baseline versus none of the placebo group (*P* = 0.045). Adverse events were higher in children who received placebo than in those administered paricalcitol during the double-blind treatment (88.9 vs. 38.9%; *P* = 0.005). In the stage 5 CKD study, eight children (61.5%) had two consecutive iPTH reductions of ≥30% from baseline, and five (38.5%) had two consecutive iPTH values of between 150 and 300 pg/mL. Clinically meaningful hypercalcemia occurred in 21% of children.

**Conclusions:**

Oral paricalcitol in children aged 10–16 years with stages 3–5 CKD reduced iPTH levels and the treatment was well tolerated. Results support an initiating dose of 1 μg paricalcitol 3 times weekly in children aged 10–16 years.

**Electronic supplementary material:**

The online version of this article (doi:10.1007/s00467-017-3579-6) contains supplementary material, which is available to authorized users.

## Introduction

Chronic kidney disease-mineral and bone disorder (CKD-MBD) is a common complication of progressive CKD [1]. Children and adults with stages 3–5 CKD frequently develop secondary hyperparathyroidism (SHPT) with an elevated intact parathyroid hormone (iPTH) level due to hypocalcemia, hyperphosphatemia, and 1,25-dihydroxycholecalciferol deficiency [2–8]. SHPT may contribute to bone disorders, vascular calcification, cardiovascular disease, and increased mortality [2, 6, 9–13]. Unlike in adults, CKD-MBD in children often results in growth retardation [14]. Serum levels of iPTH are commonly used as a noninvasive measure of CKD-MBD [15, 16].

The safety and efficacy of intermittent intravenous dosing of paricalcitol as therapy for SHPT in children with CKD undergoing dialysis have been previously demonstrated [17]. Children receiving hemodialysis treated with intravenous paricalcitol had a statistically significant decrease in iPTH levels from baseline compared with their counterparts receiving placebo (164 pg/mL decrease vs. 238 pg/mL increase, respectively; *P* = 0.03) [17]. Several studies in adults with stages 3 and 4 CKD and SHPT have demonstrated that intravenous paricalcitol efficacy in reducing iPTH is comparable with that of calcitriol, but with a reduced likelihood of serum calcium and phosphorus elevation [18, 19]. A retrospective study reported similar results in children with SHPT undergoing dialysis who were treated with intravenous paricalcitol [20].

We report here the results of two prospective, phase 3 clinical trials that were conducted to evaluate the pharmacokinetics, efficacy, and safety of oral paricalcitol in reducing iPTH levels in children with stages 3–5 CKD. These studies were part of a postmarketing requirement from the U.S. Food and Drug Administration to evaluate paricalcitol in the management of CKD in pediatric patient populations. Using dosages derived from an initial pharmacokinetic portion, the stage 3/4 CKD study further examined paricalcitol efficacy and safety in children. The stage 5 CKD study evaluated the efficacy and safety of paricalcitol capsules for the treatment of SHPT in children with stage 5 CKD undergoing hemodialysis or peritoneal dialysis.

## Methods

### Stage 3/4 CKD study

#### Study design

The stage 3/4 CKD study (NCT01020487) was conducted in two parts. Part 1 was an open-label, nonfasting, multicenter study to evaluate the pharmacokinetics of paricalcitol in 10- to 16-year-old children with stage 3 or 4 CKD, conducted at investigative sites within the USA. Blood samples for the determination of paricalcitol serum concentrations were drawn at 1, 2, 4, 6, 8, 12, 24, 36, and 48 hours following paricalcitol dosing. In Part 2, conducted at investigative sites within the USA (including Puerto Rico), Spain, Germany, Portugal, the UK, and Singapore, the safety and efficacy of oral paricalcitol for decreasing elevated serum iPTH levels in 10- to 16-year-old children with stage 3 or 4 CKD were compared with placebo. The duration of treatment was a minimum of 24 weeks. Part 2 included two treatment periods. The first was a 12-week, randomized, double-blind, placebo-controlled multicenter study in which children were randomized 1:1 to receive paricalcitol capsules or placebo. In the second treatment period, children who completed the first treatment period received open-label paricalcitol for at least 12 weeks. In Part 2, children received the study drug for a minimum of 24 weeks in total; children continued into a follow-up phase until all enrolled children had completed 24 weeks on study drug. A schedule of visits is provided in Electronic Supplementary Material (ESM) 1A.

#### Study patients

Children (male and female; including kidney transplant recipients in Part 2) were eligible for inclusion if they were 10–16 years of age and had stage 3 or 4 CKD at screening {estimated glomerular filtration rate (eGFR) 15–59 mL/min/1.73 m^2^ (calculated using the Schwartz formula based on GFR, height, and cystatin C levels) [21]}. For entry into the treatment phase, children must have had all of the following: iPTH ≥75 pg/mL (stage 3) or ≥110 pg/mL (stage 4), corrected serum calcium ≥8.4 mg/dL (2.10 mmol/L) to ≤10.2 mg/dL (2.55 mmol/L), serum phosphorus ≥2.5 mg/dL (0.81 mmol/L) to ≤5.8 mg/dL (1.87 mmol/L), and 25-hydroxyvitamin D level ≥30 ng/mL (for Part 2 only). Serum calcium was corrected for albumin [2].

For Part 2 of the study, children with a history of solid organ transplant at ≥12 months before entry into the paricalcitol treatment phase were eligible but were required to have a stable, therapeutic calcineurin inhibitor drug level, defined as having ≥2 stable levels per investigator judgment, before enrollment.

Children taking phosphate binders were required to have been taking a stable dose (consistent type and regimen) per investigator judgment, for a minimum of 4 weeks before screening. Children receiving growth hormone were required to have been receiving it for >3 months before screening and were expected to continue to receive a stable dose throughout the treatment phase.

Children with stage 3 or 4 CKD were excluded from the study if they met any of the following criteria: history of a solid organ transplant (in Part 1 only); bone marrow transplant recipient on immunosuppressant therapy; acute kidney injury within 12 weeks of screening; symptomatic or clinically significant hypocalcemia requiring active vitamin D therapy within 6 months of screening; active kidney stones within 6 months of screening; treatment with maintenance calcitonin, bisphosphonates, cinacalcet, or glucocorticoids in an equivalent dose of >5 mg prednisone daily, or other drugs known to affect calcium or bone metabolism within 4 weeks of therapy initiation; use of phosphate supplements.

Institutional Review Board approval was obtained at all participating sites before enrollment of any patients, and all participants and/or parents or legal guardians provided signed consent forms before screening.

#### Treatment

In Part 1 of the stage 3/4 CKD study, children received a single 3 μg oral dose of paricalcitol and were then assessed for intensive pharmacokinetics for 48 h. In Part 2 of the stage 3/4 CKD study, children were randomly assigned to receive double-blind paricalcitol (initial dose 1 μg) or matching placebo three times per week (TIW) for 12 weeks; children who completed the 12-week double-blind period continued to the 12-week open-label period, for a total of 24 weeks. Some children in Part 2 of the stage 3/4 CKD study continued open-label therapy beyond 12 weeks during an extension of the study period. Paricalcitol starting doses were adjusted to iPTH, not to exceed 16 μg paricalcitol TIW. Beginning at week 4, the initial paricalcitol dose could be increased in increments of 1 μg every 4 weeks based on safety observations and blood chemistry evaluations of iPTH, calcium, and phosphorus levels (see ESM 2 and 3 for dosing protocol). In general, the target iPTH range was 35–70 pg/mL for children with stage 3 CKD and 70–110 pg/mL for children with stage 4 CKD (ESM 3).

#### Study assessments

Pharmacokinetic parameters evaluated in Part 1 of the stage 3/4 CKD study included maximum observed plasma concentration (C_max_), time to C_max_ (peak time; t_max_), terminal phase elimination half-life (t_1/2_), and area under the plasma concentration–time curve (AUC_0–∞_).

The primary efficacy endpoint of the stage 3/4 CKD study was the proportion of children who achieved two consecutive ≥30% reductions from baseline in iPTH levels during the 12-week double-blind portion of the study. Secondary efficacy endpoints included the proportion of children who attained final iPTH, calcium, and phosphorus levels within the Kidney Disease Outcomes Quality Initiative (KDOQI) target ranges by CKD stage: final iPTH (stage 3: 35–69 pg/mL; stage 4: 70–110 pg/mL), calcium (age 6–12 years: 9.4–10.2 mg/dL [2.35–2.55 mmol/L]; age 13–20 years: 8.8–10.2 mg/dL [2.20–2.55 mmol/L]), and phosphorus (age 6–12 years: 3.6–5.8 mg/dL [1.16–1.87 mmol/L]; age 13–20 years: 2.3–4.5 mg/dL [0.74–1.45 mmol/L]) levels.

Additional assessments included changes in eGFR, creatinine, calcium, and phosphorus from baseline. Adverse events (AEs), changes from baseline in blood chemistry, hematology, urinary laboratory analyses, vital signs, and physical examinations were evaluated. Clinically meaningful hypercalcemia (2 consecutive corrected serum calcium values >2.55 mmol/L [10.2 mg/dL]) was also assessed and was a criterion for study discontinuation.

#### Statistical analyses

The Fisher exact test on the intent-to-treat population was used to evaluate the primary efficacy endpoint for the stage 3/4 CKD study (proportion of children who achieved 2 consecutive ≥30% reductions from baseline in iPTH levels during the 12-week double-blind period). A Cochran–Mantel–Haenszel test was used to evaluate secondary endpoints, adjusting for stage of CKD on the intent-to-treat population. Descriptive statistics were recorded for the open-label period of Part 2 of the stage 3/4 CKD study and for the safety assessment.

### Stage 5 CKD study

#### Study design

The efficacy and safety of oral paricalcitol in 10- to 16-year-old children with stage 5 CKD receiving peritoneal dialysis or hemodialysis were evaluated in a second trial (NCT01382212), conducted at investigative sites within the USA and Europe. Children received open-label paricalcitol for 12 weeks. Because of the potential health risk associated with 12 weeks of uncontrolled SHPT in children with stage 5 CKD, no placebo control group was included in the study. A schedule of visits is provided in ESM 1B. Optional study site visits at treatment weeks 6 and 10 were conducted at the discretion of the investigator to ensure safety and/or to make dose adjustments.

#### Study patients

Children (male and female) aged 10–16 years with stage 5 CKD who were receiving peritoneal dialysis or hemodialysis for ≥3 months before screening were eligible for the study. To meet eligibility criteria, children were also required to be currently diagnosed with and/or receiving treatment for SHPT. If taking phosphate binders, the patient was required to be on a stable dose (consistent type and regimen) per investigator judgment for ≥2 weeks before the start of screening.

Children receiving growth hormone were required to be taking the hormone for >3 months before screening and were expected to continue receiving a stable dose throughout the study. Children receiving vitamin D receptor (VDR) activators were required to meet inclusion and exclusion criteria before completing a 2- to 12-week washout period. VDR activator-naive children and children receiving VDR activators were required to meet all specified laboratory values in the inclusion and exclusion criteria before entering the dosing periods, namely, a corrected serum calcium level [2]; a phosphorus value of ≤6.5 mg/dL (2.1 mmol/L); an iPTH level of >300 pg/mL (31.6 pmol/L) and ≤2000 pg/mL (210.6 pmol/L).

Exclusion criteria for the stage 5 CKD study included the following: anticipated living donor kidney transplant within 3 months of treatment; kidney transplant recipient currently receiving full immunosuppressant therapy (full immunosuppressive doses of ≥2 immunosuppressant medications); anticipated dialysis discontinuation within 4 months of screening; parathyroidectomy within 12 weeks of screening; history of gastrointestinal disease, active kidney stones, or clinically significant hypocalcemia; concomitant treatment with calcitonin, bisphosphonates, glucocorticoids, cinacalcet, prescription-based phosphate supplements, or other drugs known to affect calcium or bone metabolism.

Institutional Review Board approval was obtained at all participating sites before enrollment of any patients, and all participants and/or parents or legal guardians provided signed consent forms before screening.

#### Treatment

Children in the stage 5 CKD study received paricalcitol capsules TIW (not on consecutive days) for 12 weeks. The starting dose was calculated using the following formula: iPTH (in pg/mL)/120 = μg paricalcitol (rounded down to the nearest whole number), where the iPTH value was that from the last visit before study day 1. The starting dose was limited to ≤16 μg TIW. The dose was adjusted to maintain an iPTH level of 150–300 pg/mL (15.8–31.6 pmol/L) without exceeding 16 μg TIW, based on iPTH, phosphorus, and calcium results obtained during office visits (KDOQI ranges used as target; ESM 3). Dose decreases were permitted at any time (TIW increments 2 μg). Beginning at week 4, dose increases related to iPTH levels were permitted in 4-week increments, with 1 μg TIW adjustments.

#### Study assessments

Although the stage 5 CKD study did not include formal efficacy endpoints, iPTH levels were recorded for the purposes of dose adjustments. The proportion of children who achieved two consecutive ≥30% reductions from baseline in iPTH levels and the proportion of children who achieved two consecutive iPTH levels between 150 and 300 pg/mL during the 12-week study period were assessed. AEs, changes in baseline blood chemistry, hematology, urinary laboratory analyses, vital signs, and physical examinations were evaluated. Clinically meaningful hypercalcemia (2 consecutive corrected serum calcium values of >2.55 mmol/L [10.2 mg/dL]) was also assessed.

#### Statistical analyses

The stage 5 CKD study did not include a comparator arm and, therefore, no statistical analyses were performed. Descriptive statistics were recorded for the proportion of children who achieved two consecutive ≥30% reductions from baseline in iPTH levels during the 12-week study period, the proportion of children who achieved two consecutive iPTH levels between 150 and 300 pg/mL, and the safety assessment.

## Results

### Stage 3/4 CKD study

#### Study patients

**Fig 1:**
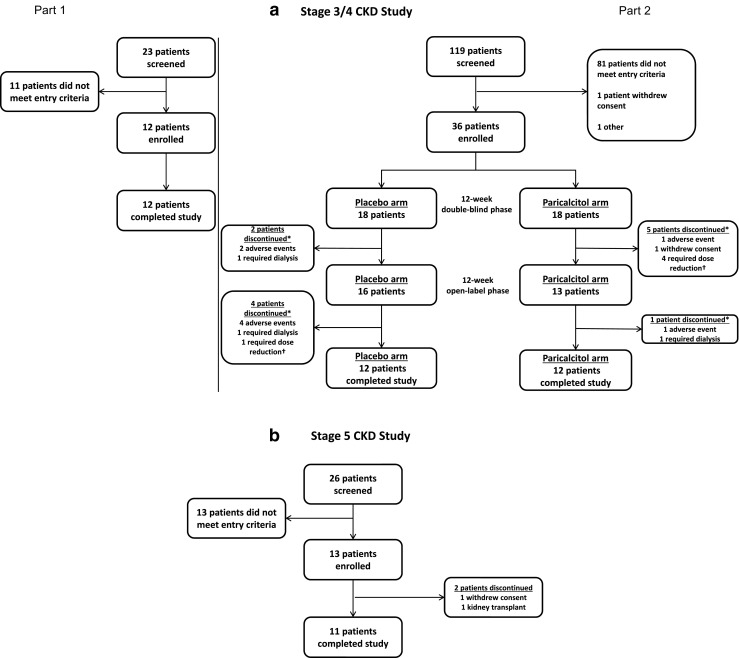
Disposition of children. CONSORT flow diagrams for the stage 3/4 chronic kidney disease (*CKD*) study (**a**) and the stage 5 CKD study (**b**). *Reasons were not mutually exclusive, ^*†*^Dose reduction to <1 μg three times per week

A total of 48 children were enrolled in the stage 3/4 CKD study (Part 1:* n* = 12; Part 2:* n* = 36). A summary of children enrolled at each investigative site is provided in ESM 4. All 12 children enrolled in Part 1 completed the pharmacokinetic study. Of the 36 children enrolled in Part 2, 80.6% (29/36) completed the double-blind phase and initiated the open-label phase, and 66.7% (24/36) completed the entire study (≥24 weeks of study drug treatment). Figure 1a provides a summary of the patient disposition for the stage 3/4 CKD study.

Demographics and baseline characteristics of children enrolled in Part 2 of the stage 3/4 CKD study are provided in Table 1. The majority of children were male and white. No statistically significant differences were observed in baseline clinical characteristics (sex, age, weight, race, and ethnicity) between children enrolled in the placebo and paricalcitol arms of Part 2. Demographics of the children enrolled in Part 1 were similar to those enrolled in Part 2 (ESM 5). Baseline vital signs and laboratory values for the intent-to-treat populations from Part 2 are summarized in Table 1. No statistically significant differences were observed in the baseline vital signs or laboratory values between the placebo and paricalcitol groups.

**Table 1:** Demographics and baseline

Characteristic	Stage 3/4 CKD Part 2				Stage 5 CKD
	Treatment group		Total study patients (*n* = 36)	*P* value	All treated
	Placebo arm (*n* = 18)	Paricalcitol arm (*n* = 18)			Paricalcitol (*n* = 13)
Sex					
Female	5 (27.8)	6 (33.3)	11 (30.6)	1.000	8 (61.5)
Male	13 (72.2)	12 (66.7)	25 (69.4)		5 (38.5)
Age (years)	13.3 ± 1.8	13.9 ± 1.8	13.6 ± 1.8	0.356	14.5 ± 1.8
Weight (kg)	48.2 ± 12.3	46.7 ± 10.2	47.4 ± 11.2	0.682	49.0 ± 19.7
Kidney transplant recipient			8 (75.0)		1 (7.7)
Race					
White	17 (94.4)	14 (77.8)	31 (86.1)	0.229	8 (61.5)
Black	0	0	0		2 (15.4)
Asian	0	3 (16.7)	3 (8.3)		1 (7.7)
American Indian/Alaska native	0	0	0		1 (7.7)
Other	1 (5.6)	1 (5.6)	2 (5.6)		0
Multirace	0	0	0		1 (7.7)^b^
Ethnicity					
Hispanic or Latino	5 (27.8)	4 (22.2)	9 (25.0)	1.000	6 (46.2)
No ethnicity	13 (72.2)	14 (77.8)	27 (75.0)		7 (53.8)
Baseline vital signs and laboratory values					
Systolic BP (mmHg)	117.06 ± 13.0	118.50 ± 14.9	117.78 ± 13.8	0.759	119.08 ± 18.9
Diastolic BP (mmHg)	67.00 ± 10.8	65.61 ± 10.9	66.31 ± 10.7	0.702	68.38 ± 14.5
Albumin (g/dL)	4.66 ± 0.37	4.52 ± 0.42	4.59 ± 0.40	0.280	4.07 ± 0.37
Serum creatinine (mg/dL)	2.33 ± 0.77	2.36 ± 0.96	2.34 ± 0.86	0.916	7.48 ± 3.15
Corrected serum calcium (mg/dL)^a^	9.86 ± 0.42	9.70 ± 0.35	9.78 ± 0.39	0.214	9.24 ± 0.68
Serum phosphorus (mg/dL)	4.44 ± 0.83	4.52 ± 0.56	4.48 ± 0.70	0.731	4.66 ± 1.13
iPTH (pg/mL)	155.44 ± 97.26	144.28 ± 64.86	149.86 ± 81.67	0.688	883.62 ± 373.81
25-Hydroxyvitamin D (ng/dL)	40.78 ± 7.90	52.17 ± 50.26	46.47 ± 35.93	0.349	18.62 ± 10.81

Data in table are presented as the mean ± standard deviation (SD) or as a number with the percentage in parentheses, as appropriate

Baseline vital signs and laboratory values were not collected for children participating in Part 1 of the stage 3/4 CKD study

BP, Blood pressure; CKD, chronic kidney disease; iPTH, intact parathyroid hormone

^a^Corrected to an albumin level of 4.0 g/dL

^b^Identified as black and white

#### Pharmacokinetics

**Fig 2:**
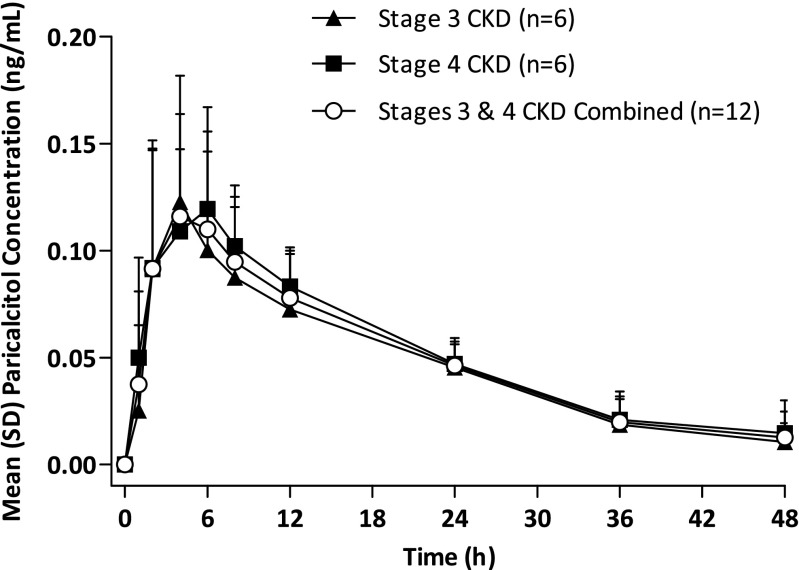
Paricalcitol plasma concentration. Mean plasma concentration-time profiles of paricalcitol. *SD* Standard deviation, *CKD* chronic kidney disease

Oral paricalcitol was quickly absorbed and reached C_max_ in plasma at approximately 4 h (Fig. 2). The pharmacokinetics of oral paricalcitol following a 3 μg dose were similar between children with stages 3 and 4 CKD (Fig. 2; Table 2).

**Table 2:** Pharmacokinetic parameters of paricalcitol in plasma

Pharmacokinetic parameter	Stage 3/4 chronic kidney disease (CKD) Part 1		Total study patients (*n* = 12)
	Treatment group		
	Stage 3 CKD (*n* = 6)	Stage 4 CKD (*n* = 6)	
t_max _(h)^a^	4.0 (4–4)	4.0 (2–8)	4.0 (2–8)
C_max_ (ng/mL)	0.12 ± 0.10	0.13 ± 0.05	0.10 ± 0.03
AUC_0–∞_ ng•h/((or ng×h/))	2.6 ± 0.8	3.2 ± 1.0	2.9 ± 0.9
t_1/2_ (h)^b^	13.3 ± 4.3	17.5 ± 5.9	14.5 ± 5.6
V/F (L)	27.8 ± 18.6	24.4 ± 5.9	26.2 ± 13.8
CL/F (L/h)	1.23 ± 0.4	1.02 ± 0.4	1.13 ± 0.4

Data in table are presented as the mean ± SD unless noted otherwise

AUC_0–∞_, Area under the curve; CL/F, oral clearance; C_max_, maximum observed plasma concentration; t_1/2_, terminal phase elimination half-life; t_max_, time to C_max_; V/F, apparent volume of distribution

^a^Median (minimum–maximum)

^b^Harmonic mean and pseudo SD

#### Efficacy and safety

During the double-blind period of the stage 3/4 CKD study, five of the 18 children (27.8%) in the paricalcitol group achieved the primary outcome of two consecutive reductions of ≥30% from baseline in iPTH levels, compared with no children from the placebo group. The between-group difference of 27.8% was statistically significant (95% confidence interval [CI] 7.5–52.8%; *P* = 0.045). The paricalcitol treatment group also experienced greater overall iPTH reduction from baseline; the mean between-group change in iPTH from baseline to final assessment was −72.4 pg/mL (95% CI −108.05 to −36.75 pg/mL; *P* < 0.001). At week 12, the mean (standard error) change from baseline in iPTH was −17.1 (19.2) for the paricalcitol group compared with +71.5 (17.7) for the placebo group. Children with stage 3/4 CKD treated with paricalcitol achieved the primary outcome in similar proportions (30 [3/10] and 25% [2/8], respectively); differences in the proportions of children who achieved the primary outcome between paricalcitol and placebo groups were not statistically significant when analyzed by individual CKD stage (stage 3 *P* = 0.090; stage 4 *P* = 0.467).

**Fig 3:**
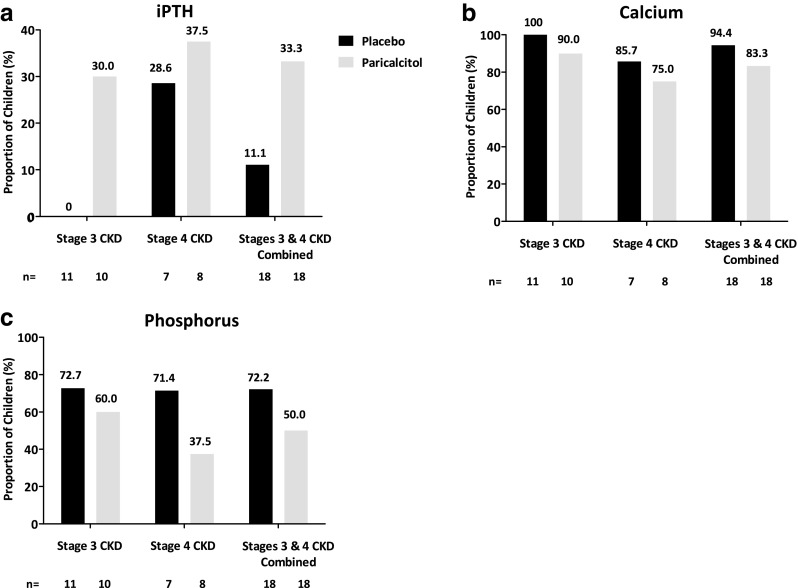
Proportion of children achieving final measures within Kidney Disease Outcomes Quality Initiative target ranges (intent-to-treat) for iPTH (**a**), calcium (**b**), and phosphorus levels (**c**). *iPTH* Intact parathyroid hormone

The proportion of children with stage 3/4 CKD achieving calcium and phosphorus levels within the KDOQI range at the final study visit was high and similar in the paricalcitol and placebo arms of the study (Fig. 3). Compared with placebo, a higher proportion of the total paricalcitol-treated population achieved final iPTH levels within the KDOQI range (33.3 [6/18] vs. 11.1% [2/18]), but this difference was not statistically significant (*P* = 0.128).

The efficacy of paricalcitol in children with stage 3/4 CKD in the open-label period was consistent with that of children in the paricalcitol treatment group from the double-blind period. Overall, of the 29 children for whom data were available, 12 (41.4%) achieved two consecutive reductions of ≥30% from baseline in iPTH levels, and eight (27.6%), 25 (86.2%), and 16 (55.2%) achieved final iPTH, calcium, and phosphorus levels within KDOQI target ranges, respectively. During the open-label period, iPTH levels decreased from baseline at week 16 (−34.4 pg/mL; 95% CI −64.9 to −3.8 pg/mL) and week 20 (−27.0 pg/mL; 95% CI −66.0 to 12.0 pg/mL), but increased by week 24 (10.1 pg/mL; 95% CI −57.4 to 77.6 pg/mL).

**Fig 4:**
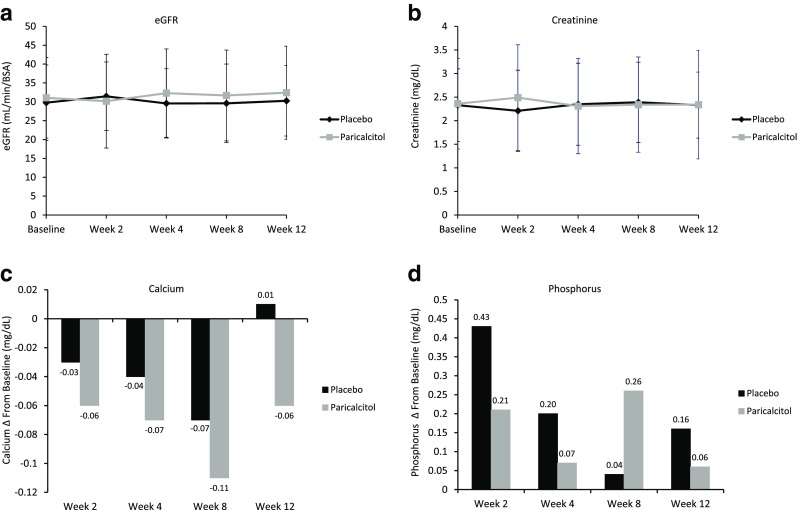
Changes in laboratory values.** a**,** b** Changes in estimated glomerular filtration rate (*eGFR*; **a**) and creatinine levels (**b**) over time.** c**,** d** Changes in calcium (**c**) and phosphorus (**d**) from baseline over time. *BSA* Body surface area

Changes in laboratory measures, including eGFR, creatinine, calcium, and phosphorus, over the 12-week double-blind period are summarized in Fig. 4. There were no statistically significant differences for any of the laboratory assessments between the paricalcitol and placebo groups at any of the time points or overall.

A summary of AEs is provided in Table 3. Treatment-emergent AEs were reported in two of the 12 children (16.7%) in Part 1, but none were severe or serious. During the double-blind period of Part 2, AEs were observed in a significantly higher proportion of children in the placebo group than in the paricalcitol group (16/18 vs. 7/18; *P* = 0.005). Two children in the placebo group experienced a serious AE compared with none in the paricalcitol group. The most common AEs were viral infection (16.7%), nasopharyngitis (11.1%), and hypercalcemia (11.1%) in the placebo group, and rhinitis (16.7%) in the paricalcitol group. One child in the paricalcitol group experienced clinically meaningful hypercalcemia per study definition (peak value 10.3 mg/dL). During the open-label period of Part 2, AEs were reported in 18 of 29 children (62.1%), of which one event was severe (3.4%) and two were serious (6.9%). Three children experienced clinically meaningful hypercalcemia and two experienced hyperphosphatemia.

**Table 3:** Summary of treatment-emergent adverse events

Treatment-emergent AE	Stage 3/4 CKD Part 1	Stage 3/4 chronic kdiney disease (CKD) Part 2						Stage 5 CKD
	All treated	Double-blind phase			Open-label phase			All treated
		Treatment group		Total study patients (*n* = 36)	Treatment group		Total study patients (*n* = 29)	
	Paricalcitol (*n* = 12)	Placebo arm (*n* = 18)	Paricalcitol am (*n* = 18)		Placebo arm (*n* = 16)	Paricalcitol arm (*n* = 13)		Paricalcitol (*n* = 13)
Any AE	2 (16.7)	16* (88.9)	7* (38.9)	23 (63.9)	12 (75.0)	6 (46.2)	18 (62.1)	11 (84.6)
Any AE possibly related to study drug^a^	0	1 (5.6)	2 (11.1)	3 (8.3)	4 (25.0)	1 (7.7)	5 (17.2)	2 (15.4)
Any severe AE^b^	0	2 (11.1)	0	2 (5.6)	0	1 (7.7)	1 (3.4)	0
Any serious AE	0	2 (11.1)	0	2 (5.6)	1 (6.3)	1 (7.7)	2 (6.9)	2 (15.4)
Any AE leading to discontinuation of study drug	0	2 (11.1)	1 (5.6)	3 (8.3)	4 (25.0)	1 (7.7)	5 (17.2)	0
Any fatal AE	0	0	0	0	0	0	0	0
Deaths^c^	0	0	0	0	0	0	0	0

*Statistically significant difference between placebo and paricalcitol at *P* = 0.005 according to the Fisher exact test

Values in table are expressed as a number with the percentage in parenthesis

AE, Adverse event

^a^Investigator assessment

^b^Defined as an adverse event with maximum severity, regardless of organ affected

^c^Includes deaths that were not treatment emergent

### Stage 5 CKD study

#### Study patients

A summary of study enrollment at investigative sites is provided in ESM 4. Of the 13 children enrolled in the stage 5 CKD study, all received one or more doses of paricalcitol and eleven (84.6%) completed the study. One child discontinued after consent was withdrawn, and one child discontinued because of a kidney transplant. Patient disposition is summarized in Fig. 1b. A summary of demographics and baseline characteristics is provided in Table 1 (last column); the majority of enrolled children were female and white. Eight patients reported receiving hemodialysis and seven received peritoneal dialysis, among whom two reported receiving both hemodialysis and peritoneal dialysis. Baseline vital signs and laboratory values for the intent-to-treat population are summarized in Table 1 (last column).

#### Efficacy and safety

In the stage 5 CKD study, five of the 13 children (38.5%) had two consecutive iPTH values between 150 and 300 pg/mL and eight (61.5%) had two consecutive iPTH reductions of ≥30% from baseline. All 13 children were treated with paricalcitol; 11 (84.6%) experienced an AE and two (15.4%) experienced a serious AE (Table 3; last column). Overall, 23% of children experienced clinically meaningful hypercalcemia (defined as 2 consecutive serum calcium values of >10.5 mg/dL) with paricalcitol during the study.

**Fig 5:**
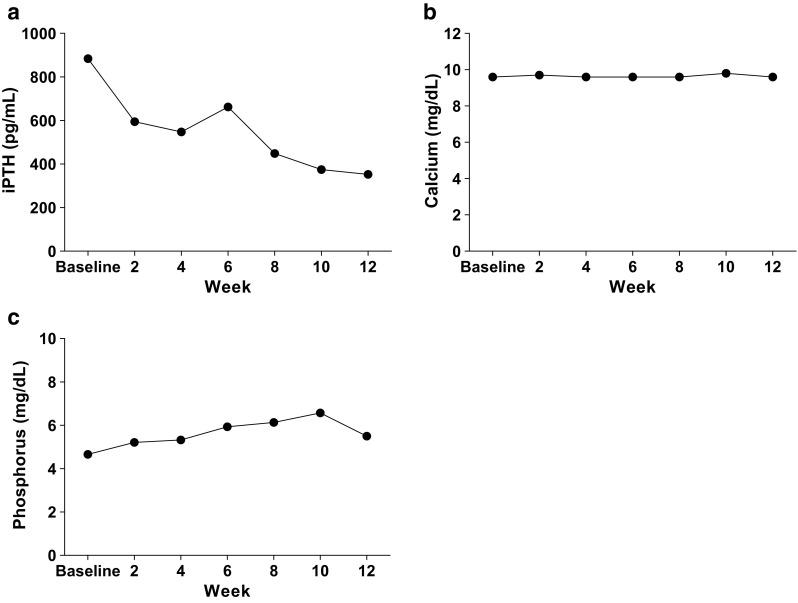
Changes in intact parathyroid hormone (*iPTH*;** a**), calcium (**b**), and phosphorus (**c**) over time in patients with stage 5 chronic kidney disease

iPTH, calcium, and phosphorus levels over the 12-week double-blind period in patients with stage 5 CKD are shown in Fig. 5. With the exception of the expected decrease in iPTH with paricalcitol, there were minor changes in calcium and phosphorus over the 12-week period.

## Discussion

Although CKD differs in adults and children, the pathophysiology of SHPT, which is characterized by elevated iPTH levels and parathyroid gland hyperplasia, is an early and common complication regardless of age group [2–8]. The occurrence of elevated iPTH levels is the major contributor to the high-turnover bone disease seen in both adults and children and to the clinical symptoms of extraskeletal calcification, bone pain, and fractures [1]. Unlike in adults, the complications of bone disease in children with CKD also manifest clinically as growth retardation and musculoskeletal deformities [14].

Clinical studies in adult patients with stage 3 or 4 CKD have shown that paricalcitol capsules significantly reduce iPTH levels, with no significant difference between paricalcitol capsules and placebo in terms of incidences of hypercalcemia, hyperphosphatemia, and elevated calcium–phosphorus product [22]. However, limited clinical data are available for the use of paricalcitol in children with SHPT associated with CKD.

In our study, C_max_, AUC_0–∞_, t_max_, and t_1/2_ values for paricalcitol appeared to be similar in children with stage 3 or 4 CKD. The exposure (AUC) of paricalcitol in children with stage 3 or 4 CKD was numerically higher than that observed in adults with stage 3 or 4 CKD [22]. Based on prospectively defined criteria for the selection of the paricalcitol dose for Part 2 of the stage 3/4 CKD study, we selected a dose of 1 μg paricalcitol TIW as the initial dose in the efficacy and safety portion of the study, and the results support an initiating dose of 1 μg paricalcitol TIW in future paricalcitol studies in children aged 10–16 years, as well as in clinical practice. The population pharmacokinetic analysis shows that the pharmacokinetics of paricalcitol in pediatric patients with stage 5 CKD appeared to be similar to those observed in pediatric patients with stage 3 or 4 CKD (data on file). These analyses indicate that renal function does not impact paricalcitol exposure in pediatric patients, which is also consistent with the findings in adults [22].

Paricalcitol was effective in reducing iPTH levels in the children with stage 3 or 4 CKD enrolled in our study, and there was a statistically significantly greater proportion of children who achieved two consecutive reductions of ≥30% from baseline in iPTH levels with paricalcitol treatment compared with placebo. Overall, there was a greater reduction in iPTH levels from baseline to final assessment in the paricalcitol treatment group than in the placebo arm. The fact that most children enrolled in the study did not have severe SHPT and had well-controlled mineral metabolism may explain the observed moderate decrease in iPTH. The effect of paricalcitol treatment was sustained in children with stage 3 or 4 CKD for approximately 20 weeks. The decrease in suppression of iPTH observed at week 24 in the open-label study supports ongoing monitoring of iPTH levels in children treated with paricalcitol. Adjustment of paricalcitol dosage to maintain reductions in iPTH levels should take into account concurrent calcium and phosphorus levels.

In the stage 3/4 CKD study, there was no difference between treatment groups in the proportion of children who achieved final iPTH, calcium, or phosphorus levels within KDOQI targets. Changes in laboratory values were also similar between the paricalcitol and placebo groups. The iPTH results suggest that a larger study population may have yielded a significant difference in the achievement of KDOQI target range between groups. A high proportion of children in the placebo group achieved calcium and phosphorus levels within KDOQI ranges, suggesting that a significant improvement with paricalcitol treatment may be unlikely. Both eGFR and creatinine levels remained stable throughout the course of the 12-week double-blind portion of the stage 3/4 CKD study, suggesting that renal function was not significantly affected by paricalcitol treatment.

Consistent with previous studies, hypercalcemia events with paricalcitol treatment were rare [17, 19, 23]. Overall, paricalcitol was well tolerated in children with stages 3–5 CKD. Most AEs were mild, with few serious or severe events, and no new clinically concerning safety signals were observed in either study.

The results presented herein are consistent with those of Greenbaum et al. [17] who demonstrated the efficacy of intravenous paricalcitol. Previous studies have demonstrated that calcitriol and doxercalciferol exhibit efficacy in lowering PTH levels and help to control bone turnover [24, 25]; however, it has also been reported that intermittent high-dose calcitriol may adversely affect linear bone growth [26]. Paricalcitol is effective in reducing iPTH levels without typically elevating the levels of calcium and phosphorus and may have an even greater potential to control bone turnover while minimizing the risk of adverse effects on linear bone growth. Future studies will be required to evaluate the efficacy of paricalcitol in controlling bone turnover.

Both studies share the limitations inherent in trials of short duration or limited number of patients that seek to determine differences in outcome measurements and identify potential safety concerns. Despite the limited number of patients, completion rates in the stage 3/4 CKD study (80.5% in the double-blind phase) and stage 5 CKD study (84.6%) were high compared with other studies of this type [17, 23]. A larger and longer study may have allowed for detection of differences in efficacy and safety between children with stage 3 and stage 4 CKD. It is also possible that larger studies of longer duration would have increased the potential for observation of infrequent AEs. Inclusion of children with stage 3 CKD who were vitamin D replete may have limited the potential to markedly reduce iPTH levels in the study population. In children with stage 3 CKD with adequate levels of calcifediol, active vitamin D supplementation may not be necessary to manage secondary hyperparathyroidism [3].

In summary, data from these studies demonstrate that the treatment of children aged 10–16 years with stages 3–5 CKD with oral paricalcitol was effective in lowering iPTH levels and was well tolerated over extended trial periods. Additionally, the administration of paricalcitol capsules for the treatment of SHPT in pediatric patients aged 10–16 years with stage 3 or 4 CKD appears to be similar to that observed in adult patients. These results support an initiating dose of 1 μg paricalcitol TIW in future paricalcitol studies in children 10–16 years of age with CKD, as well as in clinical practice. Future studies will need to evaluate dosing, efficacy, and safety of oral paricalcitol in younger children with CKD.

## Electronic supplementary material

Below is the link to the electronic supplementary material.

ESM 1 (DOCX 238 kb)
