# Supplementary material for: Efficacy and safety of paricalcitol in children with stages 3 to 5 chronic kidney disease
Source: Pediatr Nephrol. 2017 Mar 22;32(7):1221–32. doi: 10.1007/s00467-017-3579-6 (PMC5440538; doi:10.1007/s00467-017-3579-6)
Supplement: Supplementary file 1 — (DOCX 238 kb) [file 467_2017_3579_MOESM1_ESM.docx]

**Supplementary Material**

***Paricalcitol Is Effective and Well Tolerated in Children With Stages 3 to 5 Chronic Kidney Disease***

**Pediatric Nephrology**

**Authors:** Nicholas J. A. Webb,^1,*^ Gary Lerner,^2^ Bradley A. Warady,^3^ Katherine M. Dell,^4^ Larry A. Greenbaum,^5^ Gema Ariceta,^6^ Bernd Hoppe,^7^ Peter Linde,^8^ Ho-Jin Lee,^8^ Ann Eldred,^8^ Matthew B. Dufek^8^

^1^Department of Paediatric Nephrology and NIHR/Wellcome Trust Clinical Research Facility, University of Manchester, Manchester Academic Health Science Centre, Royal Manchester Children’s Hospital, Manchester, UK; ^2^Keck School of Medicine, Pediatric Nephrology, Children’s Hospital Los Angeles, Los Angeles, CA, USA; ^3^Division of Pediatric Nephrology, Children’s Mercy Hospital, Kansas City, MO, USA; ^4^Case Western Reserve University and Center for Pediatric Nephrology, Cleveland Clinic, Cleveland, OH, USA; ^5^Emory School of Medicine and Children’s Healthcare of Atlanta, Atlanta, GA, USA; ^6^Pediatric Nephrology, University Hospital Vall d’Hebron, Universitat Autonoma de Barcelona, Barcelona, Spain; ^7^University Hospital Bonn, Bonn, Germany; ^8^AbbVie Inc., North Chicago, IL, USA

***Address correspondence to:**

Nicholas J. A. Webb, DM, FRCP, FRCPCH

Royal Manchester Children’s Hospital

Oxford Road

Manchester, M13 9WL, UK

nicholas.webb@cmft.nhs.uk

**Online Resource 1.** Study schematics for (A) Part 2 of the stage 3/4 CKD study and (B) the stage 5 CKD study. ET, early termination; FUWO1, FUWO2, FUWO3, follow-up washout visits 1, 2, 3; S1, S2, S3, screening visits 1, 2, 3; VDR, vitamin D receptor; WO, washout. *Patients to be rescreened up to 2 times with ≥2 weeks between rescreening attempts. ^†^Patients to be enrolled into the next study period within 2 weeks of the applicable qualifying visit. ^‡^Optional visit per investigator’s discretion to ensure child safety and/or for dose adjustment purposes

**A**


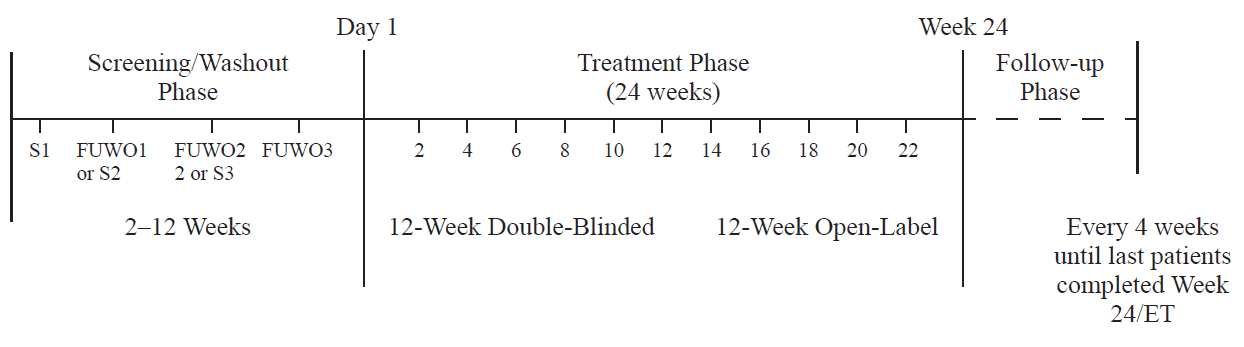


**B**


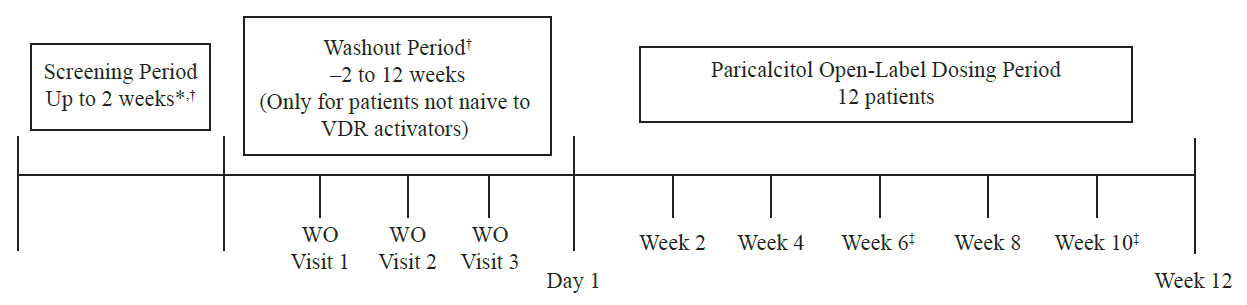


**Online Resource 2.** Paricalcitol dose selection protocol for the (A) stage 3 and (B) stage 4 CKD study. Ca, calcium; iPTH, intact parathyroid hormone; P, phosphorus

**Stage 3 CKD**


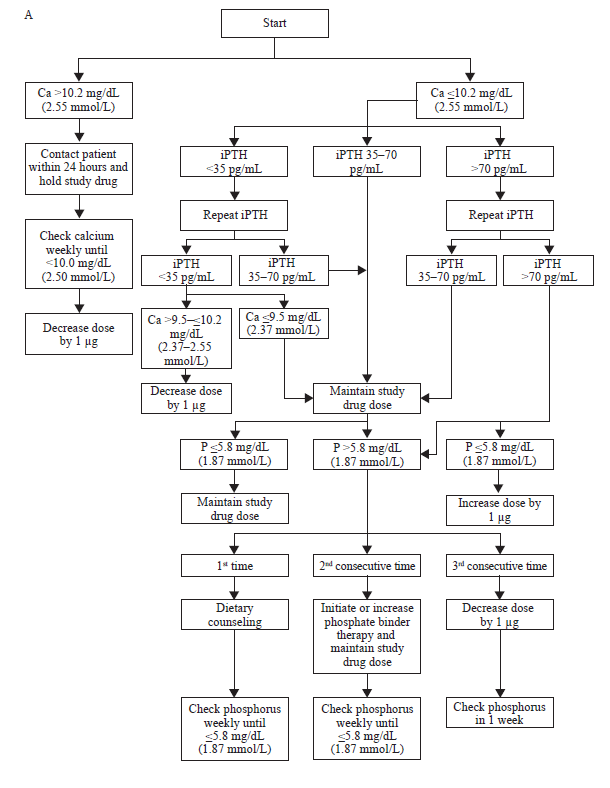


**Stage 4 CKD**


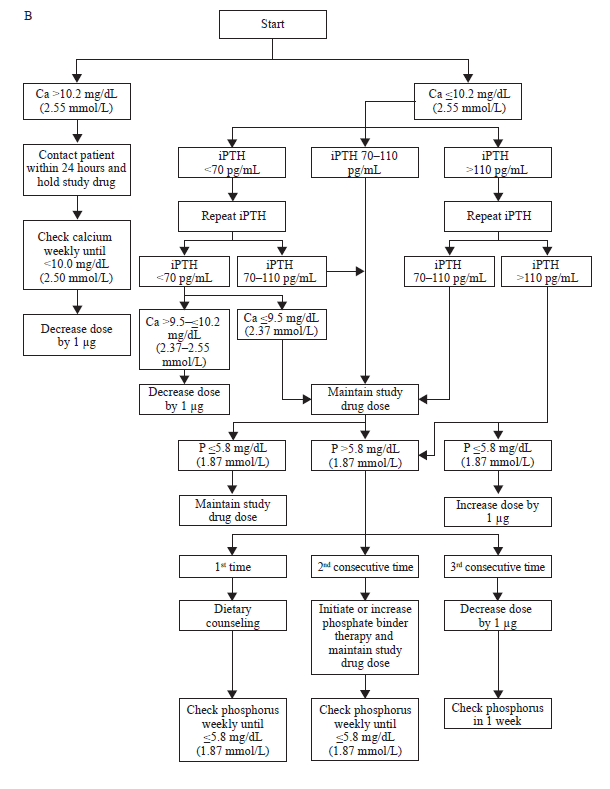


**Online Resource 3. Paricalcitol Dose Adjustment Protocol for the Stage 3/4 CKD Study**

**Dosing Decisions for iPTH Levels Stage 3 CKD**

● If iPTH was >70 pg/mL with calcium (adjusted) ≤10.2 mg/dL (2.55 mmol/L) and phosphorus ≤5.8 mg/dL (1.87 mmol/L), then the patient was to return at the next scheduled visit to undergo a limited chemistry evaluation

○ If the repeated iPTH was >70 pg/mL with calcium (adjusted) ≤10.2 mg/dL (2.55 mmol/L) and phosphorus ≤5.8 mg/dL (1.87 mmol/L), then the dose of study drug was to be increased by 1 μg TIW

● If iPTH was ≥35 pg/mL and ≤70 pg/mL with calcium ≤10.2 mg/dL (2.55 mmol/L) and phosphorus ≤5.8 mg/dL (1.87 mmol/L), then study drug was to be maintained at the current dose

● If iPTH was <35 pg/mL, then the patient was to return at the next scheduled visit to undergo a limited chemistry evaluation

○ If the repeated iPTH was <35 pg/mL, AND

- If serum calcium was >9.5 mg/dL (2.37 mmol/L) and <10.2 mg/dL (2.55 mmol/L), then the dose of study drug was to be reduced by 1 μg TIW. The patient was to return in 2 weeks for a repeat PTH measurement; OR
- If serum calcium was ≤9.5 mg/dL (2.37 mmol/L), then study drug was to be maintained at the current dose

**Dosing Decisions for iPTH Levels Stage 4 CKD**

● If iPTH was >110 pg/mL with calcium (adjusted) ≤10.2 mg/dL (2.55 mmol/L) and phosphorus ≤5.8 mg/dL (1.87 mmol/L), then the patient was to return at the next scheduled visit to undergo a limited chemistry evaluation

○ If the repeated iPTH was >110 pg/mL with calcium (adjusted) ≤10.2 mg/dL (2.55 mmol/L) and phosphorus ≤5.8 mg/dL (1.87 mmol/L), then the dose of study drug was to be increased by 1 μg TIW

● If iPTH was ≥70 pg/mL and ≤110 pg/mL with calcium ≤10.2 mg/dL (2.55 mmol/L) and phosphorus ≤5.8 mg/dL (1.87 mmol/L), then the study drug was to be maintained at the current dose

● If iPTH was <70 pg/mL, then the patient was to return at the next scheduled visit to undergo a limited chemistry evaluation

○ If the repeated iPTH was <70 pg/mL, AND

- If serum calcium was >9.5 mg/dL (2.37 mmol/L) but <10.2 mg/dL (2.55 mmol/L), then the dose of study drug was to be reduced by 1 μg TIW; the patient was to return in 2 weeks for a repeat PTH measurement, OR
- If serum calcium was ≤9.5 mg/dL (2.37 mmol/L), then study drug was to be maintained at the current dose

**Dosing Decisions for Calcium Levels Stage 3 and Stage 4 CKD**

If at any time adjusted calcium was assessed to be >10.2 mg/dL (2.55 mmol/L)

● Site was to make every effort to contact the patient within 24 hours, and instruct him or her to hold their dose of study drug

● Starting within 2 weeks (at an unscheduled visit), the site was to check serum calcium value weekly until it was observed to be <10.0 mg/dL (2.5 mmol/L)

● Restarting or discontinuing study medication was dependent on the following conditions:

○ For a patient receiving 1 μg TIW of study drug

- If calcium was observed to be ≤10.2 mg/dL (2.55 mmol/L) within 2 weeks, the patient may have resumed 1 μg TIW dose of study drug
- If the calcium was >10.2 mg/dL (2.55 mmol/L) after 2 weeks, the patient was to be discontinued from treatment and withdrawn from the study

○ For a patient receiving ≥2 μg TIW

- When calcium returned to ≤10.2 mg/dL (2.55 mmol/L), then study drug was to be restarted at a dose 1 μg TIW lower than the previous dose; for example, if patient was receiving 2 μg TIW when elevated calcium was observed, study drug was to be restarted at 1 μg TIW

● If calcium remained elevated, the study drug was to be discontinued and the patient withdrawn from the study

**Dosing Decisions for Phosphorus Levels Stage 3 and Stage 4 CKD**

● At the first occurrence of phosphorus >5.8 mg/dL (1.87 mmol/L), the patient was to receive dietary counseling

● At the second consecutive occurrence of phosphorus >5.8 mg/dL (1.87 mmol/L), phosphate binder therapy was to be initiated for the patient or modified for patients already having phosphate binder therapy

● At the third consecutive occurrence of phosphorus >5.8 mg/dL (1.87 mmol/L), the dose of study drug was to be reduced by 1 μg TIW

○ If the patient was receiving 2 μg TIW, then the dose was to be decreased to 1 μg TIW

○ If the patient was receiving 1 μg TIW, then the study drug was to be discontinued and the patient withdrawn from the study

Throughout the Safety and Efficacy Portion, Part 2 of the study, if a patient receiving a 2-μg dose TIW required dose reduction for any reason, then the dose was to be restricted to 1 μg TIW taken no more frequently than every other day. If a patient required a dose reduction below 1 μg TIW, the study drug was to be discontinued and the patient withdrawn from the study.

**Paricalcitol Dose Adjustment Protocol for the Stage 5 CKD Study**

**Dosing Decisions for iPTH Levels**

Dose was to be adjusted according to the following criteria based on iPTH levels:

● If iPTH level was >300 pg/mL and corrected calcium level was ≤10.2 mg/dL (2.55 mmol/L) and phosphorus level was ≤6.5 mg/dL (2.1 mmol/L)

○ The dose of study drug was to be increased by 1 μg TIW or restarted at a dose of iPTH/120 if the dose was previously being withheld

○ The patient was to return at the next scheduled study visit

● If iPTH level was ≥150 pg/mL and ≤300 pg/mL and corrected calcium level was ≤10.2 mg/dL (2.55 mmol/L) and phosphorus level was ≤6.5 mg/dL (2.1mmol/L)

○ The dose of study drug was to be maintained at the current dose or restarted at a dose of iPTH/120 if dose was previously being withheld

○ The patient was to return at the next scheduled study visit

● If iPTH level was <150 pg/mL and corrected calcium level was ≤10.2 mg/dL (2.55 mmol/L) and phosphorus level was ≤6.5 mg/dL (2.1 mmol/L)

○ The dose of study drug was to be decreased by 2 μg TIW; if the patient was receiving a dose of 1 or 2 μg TIW, then the dose was to be withheld

○ iPTH level was to be rechecked at the next scheduled study visit until iPTH level reached ≥150 pg/mL and ≤300 pg/mL

○ If iPTH level was still <150 pg/mL upon being rechecked, then the dose was to be further decreased; if the patient was receiving a dose of 1 or 2 μg TIW, then the dose was to be withheld

○ If patient’s dose was withheld for more than 2 consecutive study visits, the patient was to be discontinued from study drug; however, the investigator was to recheck iPTH level weekly until the iPTH level normalized (≥150 pg/mL and ≤300 pg/mL)

**Dosing Decisions for Calcium Levels**

If at any time the corrected calcium level was >10.2 mg/dL (2.55 mmol/L), then the appropriate action from the following list was to be applied:

● The dose of study drug was to be reduced by 2 μg TIW. If the patient was receiving 1 or 2 μg TIW, then the dose was to be withheld

● The corrected calcium level was to be rechecked weekly until it reached ≤10.2 mg/dL (2.55 mmol/L); the dose reduction was to be repeated if the corrected calcium level was still >10.2 mg/dL (2.55 mmol/L); if the corrected calcium level reached ≤10.2 mg/dL (2.55 mmol/L), then the investigator was to proceed to the dosing decisions for iPTH levels

● If patient’s dose was withheld for 2 consecutive weeks, the patient was to be discontinued from study drug; however, the investigator was to recheck corrected calcium level weekly until the corrected calcium levels normalized (≤10.2 mg/dL [2.55 mmol/L])

**Dosing Decisions for Phosphorus Levels**

If at any time phosphorus results were >6.5 mg/dL (2.1 mmol/L), phosphorus was to be checked weekly (per investigator’s discretion) until levels reached ≤6.5 mg/dL (2.1 mmol/L) and the appropriate action from the following list was to be applied:

● First occurrence of phosphorus >6.5 mg/dL (2.1 mmol/L) by a patient

○ Patient was to receive dietary counseling (per investigator discretion)

○ Study drug dose was to be maintained

● Second consecutive occurrence of phosphorus >6.5 mg/dL (2.1 mmol/L) by a patient

○ Phosphate binder therapy was to be initiated, increased, or modified (per investigator discretion)

○ Study drug dose was to be maintained

● Third consecutive occurrence of phosphorus >6.5 mg/dL (2.1 mmol/L) by a patient

○ Dose of study drug was to be decreased by 2 μg TIW

○ If patient was receiving 1 or 2 μg TIW, then the dose was to be withheld

● After the third consecutive occurrence of a phosphorus level >6.5 mg/dL (2.1 mmol/L) by a patient, phosphorus level was to be rechecked weekly until it returned to ≤6.5 mg/dL (2.1 mmol/L)

○ If the patient’s dose had been withheld for 2 consecutive weeks, the patient was to be discontinued from study drug

○ Regardless of dose administration, the investigator was to recheck phosphorus levels weekly until it normalized at ≤6.5 mg/dL (2.1 mmol/L)**Online Resource 4. Site Enrollment for the Stage 3/4 and Stage 5 CKD Studies**

| **Patients Enrolled (ITT Population) Per Investigative Site, n** | **CKD Stage 3/4 Study**  **Part 1** | **CKD Stage 3/4 Study  Part 2** | **CKD Stage 5 Study** |
| --- | --- | --- | --- |
| Germany | 0 | 3 | 0 |
| Portugal | 0 | 5 | 1 |
| Singapore | 0 | 1 | 0 |
| Spain | 0 | 6 | 0 |
| United Kingdom | 0 | 3 | 1 |
| United States | 12 | 18 | 11 |

CKD, chronic kidney disease; ITT, intent-to-treat.

**Online Resource 5. Demographics and Baseline Characteristics**

|  | **Stage 3/4 Part 1** | | | |
| --- | --- | --- | --- | --- |
|  | **CKD Stage** | |  |  |
| **Characteristic** | **Stage 3 n=6** | **Stage 4 n=6** | **Total N=12** | ***P* Value** |
| Sex, n (%) |  |  |  |  |
| Female | 1 (16.7) | 2 (33.3) | 3 (25.0) | 1.000 |
| Male | 5 (83.3) | 4 (66.7) | 9 (75.0) |  |
| Age, y, mean ± SD | 13.8±2.1 | 13.2±1.9 | 13.5±2.0 | 0.584 |
| Weight, kg, mean ± SD | 54.0±27.6 | 46.8±11.9 | 50.4±20.6 | 0.572 |
| Race, n (%) |  |  |  |  |
| White | 6 (100) | 4 (66.7) | 10 (83.3) | — |
| Black | 0 | 1 (16.7) | 1 (8.3) |  |
| Asian | 0 | 0 | 0 |  |
| American Indian/Alaska native | 0 | 1 (16.7) | 1 (8.3) |  |
| Native Hawaiian or  other Pacific Islander | 0 | 0 | 0 |  |
| Other | 0 | 0 | 0 |  |
| Multirace | 0 | 0 | 0 |  |
| Ethnicity, n (%) |  |  |  |  |
| Hispanic or Latino | 3 (50.0) | 1 (16.7) | 4 (33.3) | 0.545 |
| No ethnicity | 3 (50.0) | 5 (83.3) | 8 (66.7) |  |

CKD, chronic kidney disease.
